# Guy’s Hospital Reports

**Published:** 1887-04

**Authors:** 


GUY'S HOSPITAL REPORTS. *By* FREDERICK TAYLOR, M.D., *and* N. DAVIES-COLLEY, M.A., M.C. *Vol. XLIII, 1885–6, pp. 514. London:* J. & A. CHURCHILL, *New Burlington Street, 1886.*

This volume contains numerous plates, woodcuts and other illustrations. The record of the observations of special importance occurring during the year in so large a hospital as Guy's, cannot fail to be one of great interest, both to general and special practitioners of medicine.

The article on " The Pathology of Extravasation of Urine and of Sacculation of the Urethra and Bladder" condemns as incorrect some of the statements on the subject made by Sir Henry Thompson in Holmes' System of Surgery, and the opinions advanced are supported by illustrative cases, the post mortem conditions of which are carefully described. The article on "Uterine Dilatation" supports the views expressed in the last edition of Barnes' "System of Obstetric Medicine and Surgery," and presents many cases in illustration. The writer of the article, "On the Changes in the Vascular System in Bright's Disease," strongly favors "the view that a simple nephritis is a competent cause of these special morbid changes." Many of the other articles in the volume are as interesting as those referred to, but the limits of this notice prevent enumeration or special mention of them.

